# Physiological cold tolerance evolves faster than climatic niches in plants

**DOI:** 10.3389/fpls.2023.1257499

**Published:** 2023-09-08

**Authors:** Yin Wen, Qing Ye, Cristian Román-Palacios, Hui Liu, Guilin Wu

**Affiliations:** ^1^ Key Laboratory of Vegetation Restoration and Management of Degraded Ecosystems, Guangdong Provincial Key Laboratory of Applied Botany, South China Botanical Garden, Chinese Academy of Sciences, Guangzhou, China; ^2^ College of Life Sciences, Gannan Normal University, Ganzhou, China; ^3^ School of Information, University of Arizona, Tucson, AZ, United States

**Keywords:** physiological tolerance, climatic niche, climate change, evolution, heat tolerance

## Abstract

Understanding how plants respond to thermal stress is central to predicting plant responses and community dynamics in natural ecosystems under projected scenarios of climate change. Although physiological tolerance is suggested to evolve slower than climatic niches, this comparison remains to be addressed in plants using a phylogenetic comparative approach. In this study, we compared i) the evolutionary rates of physiological tolerance to extreme temperatures with ii) the corresponding rates of climatic niche across three major vascular plant groups. We further accounted for the potential effects of hardening when examining the association between physiological and climatic niche rates. We found that physiological cold tolerance evolves faster than heat tolerance in all three groups. The coldest climatic-niche temperatures evolve faster than the warmest climatic-niche temperatures. Importantly, evolutionary rates of physiological cold tolerance were faster than rates of change in climatic niches. However, an inverse association between physiological cold tolerance and responding climatic niche for plants without hardening was detected. Our results indicated that plants may be sensitive to changes in warmer temperatures due to the slower evolutionary rates of heat tolerance. This pattern has deep implications for the framework that is being used to estimate climate-related extinctions over the upcoming century.

## 1 Introduction

Climate change (average 0.015°C/year in 1959–2015) (Glikson, 2016) is occurring faster than it did in any other geological time in the past (the maximum of 0.0015°C/year in the Palaeocene–Eocene thermal maximum [PETM]) (Zeebe et al., 2009). In principle, species exposed to stressful conditions could potentially avoid climate-related extinctions over the upcoming decades by either 1) dispersing to areas with more suitable climatic conditions or 2) shifting their niche through plastic/adaptive responses. However, previous studies have suggested that dispersal rates (latitudinal 16.9 km/decade, elevational 11 m/decade) are much slower than the ongoing rates of climate change (Chen et al., 2011). Therefore, it is reasonable to suggest that many plant species will still need to shift their niches, instead of dispersing, in order to avoid experiencing climate-related extinctions (Corlett and Westcott, 2013). Thus, understanding the specific mechanisms that underlie niche shifts bears special importance in predicting plant species responses under projected climate change.

Plant thermal tolerance, generally understood from physiological tolerance measurements or approached from the description of climatic niches, can be used to understand spatial and temporal patterns of species’ survival and growth. Physiological tolerance, including plant photosynthetic cold/heat tolerance, is derived from the physiological response curves to temperature changes. These physiological descriptors are usually calculated using relatively short-term experimental conditions (Geange et al., 2021). Conversely, climatic niches are calculated from climate variables across species’ geographical ranges and tend to long-term adaptation to climatic conditions (Colwell and Rangel, 2009). Climatic niche reflects the complex tolerance to thermal and water stress, as well as to a single physiological process (Bush et al., 2018). Therefore, shifts in thermal tolerance can be achieved through either physiological or climatic niche shifts. For example, congeneric species distributed in contrasting climatic zones are expected to evolve divergent physiological tolerance, such as the temperature at 50% loss of photosynthesis rate under cold stress, to adapt to local climates by enhancing the stress resistance directly (Armstrong et al., 2020). In contrast, the niche expansion of angiosperms into freezing areas is associated with deciduous phenology, herbaceous growth form, and narrow vessel conduit (Zanne et al., 2014). These growth form or phenology changes avoid (e.g., deciduous phenology and short life cycle take advantage of seasons with optimum temperatures) or indirectly strengthen (e.g., conduit narrowing decreases the potential for freezing/thawing-induced embolism) the freezing tolerance of plants, which, in turn, expand their climatic niche.

To our knowledge, only a few studies have estimated evolutionary rates of thermal tolerance in plant lineages, especially based on physiological tolerance measurements (Araujo et al., 2013; Jezkova and Wiens, 2016; Lancaster and Humphreys, 2020; Liu et al., 2020b; Perez and Feeley, 2020). However, no study has explicitly compared evolutionary rates of physiological tolerance and corresponding rates of climatic niches in plants. For instance, a recent study focused on both plants and animals suggested that the evolutionary rate of the coldest temperatures in the climatic niche was higher than that of the hottest temperatures (Liu et al., 2020b). Congruently, another study using a large database of physiological tolerance for 1,028 plant species found that evolutionary rates of cold tolerance are much faster than heat tolerance, although paired comparison of the two tolerances was not performed in the study (Lancaster and Humphreys, 2020). Nevertheless, despite the obvious constraints that physiological tolerance is expected to impose on climatic niches, previous studies have not compared the evolutionary rates between physiological tolerance and climatic niche in plants. In fact, although studies on the association between rates of climatic niche and physiological tolerance have been conducted in animals (Qu and Wiens, 2020), the relationship between climatic niche and physiological tolerance remains largely unexplored in plants. Given that physiological tolerance contributes to species’ climatic niches and that the evolution of climatic niches should lag behind in the evolution of physiological tolerance, we suggest that evolutionary rates of physiological tolerance are faster than the responding climatic niche in plant species.

The association between rates of climatic and physiological evolution in plants is likely mediated by additional factors. For instance, hardening is a form of plasticity that physiological tolerance enhances after a pre-experience of stress. Exposure to stress (e.g., extreme temperature) is expected to have consequences on plant structure and function, which, in turn, would alter the physiological tolerance (Buchner et al., 2017; Janmohammadi et al., 2018; Armstrong et al., 2020). Therefore, hardening should increase or decrease the physiological tolerance value, which consequently is expected to affect the ancestral state reconstructions and evolutionary rate estimates of physiological tolerance. For instance, Lancaster and Humphreys (2020) found that the estimates of evolutionary rates are higher in hardened than non-hardened plants for both cold and heat physiological tolerance (Lancaster and Humphreys, 2020). Despite the potential hardening plasticity for physiological tolerance, the climatic niche of a species would keep stable during a relatively long time period of decades of years (Liu et al., 2020a). The uncertain change of rate in hardening status may obscure the rate comparison between climatic niche and physiological tolerance. Therefore, we suggest that the hardening condition should influence the association of the comparison between climatic niche and physiological tolerance.

We compiled a dataset of variables related to plant physiological tolerance to test 1) whether evolutionary rates of physiological tolerance are faster than the responding climatic niche in plant species and 2) if hardening affects the association between climatic niche and physiological tolerance. We calculated the climatic niche of each species based on their native geographic distribution. We performed phylogenetic comparative analyses to calculate the overall evolutionary rates of physiological tolerance and climatic niches in both heat and cold tolerance to test our hypothesis that 1) evolutionary rates of physiological tolerance are higher than the responding climatic niche in plants and 2) hardening status would influence the results of the comparison between climatic niche and physiological tolerance.

## 2 Materials and methods

### 2.1 Data collection

Physiological tolerance data of plants were compiled from different published articles and books (**Table S1**), but primarily from the GlobTherm database (Bennett et al., 2018) and Lancaster and Humphreys (2020). Note that Lancaster and Humphreys (2020) focused on the global variation of plant physiological tolerance and provided insights into the sources of variation by focusing on local environments, phylogeny, and biogeographic histories. However, our study expands on this original analytical framework by conducting paired comparisons between the evolutionary rate of physiological tolerance and the corresponding climatic niche.

**Figure 1:**
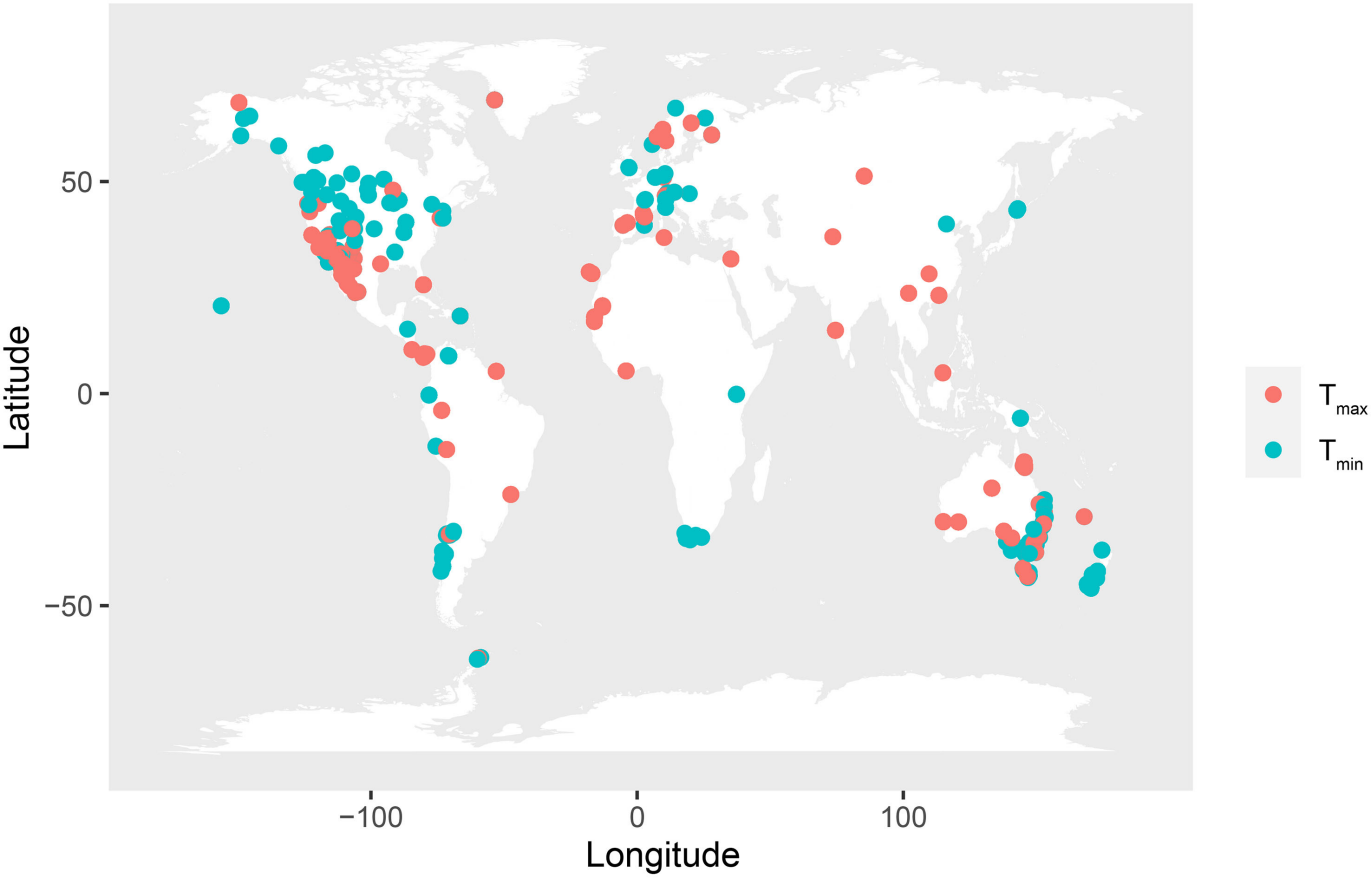
Global distribution of the physiological heat tolerance (T_max_, red circle, 1,271 records) and cold tolerance (T_min_, green circle, 882 records) records analyzed in this study. Each point indicates one site on the map.

Study sites of the physiological measurements covered warm-to-cold areas across the globe for both heat tolerance and cold tolerance (**Figure 1**). To maximize the taxonomic breadth of our dataset, multiple traits for cold and heat physiological tolerance were included in our dataset, such as freezing resistance (FR), freezing temperature (FT), temperature at 50% leak (LT_50_), and temperature at 100% leak (LT_100_) for cold tolerance and temperature at 50% leak (LT_50_), temperature at 100% leak (LT_100_), maximum temperature at photosynthetic and respiratory machinery can function (T_max_), and critical temperature (CT) for heat tolerance. Data for physiological cold tolerance were summarized in our dataset as T_min_ and physiological heat tolerance as T_max_. Although different approaches reflect different mechanisms to estimate species’ physiological tolerance values, alternative measurements tend to be strongly correlated (O'sullivan et al., 2013; O'sullivan et al., 2016). The use of physiological tolerance values derived from different approaches is not expected to significantly affect the big picture of rate comparison (**Table S2**).

Following Lancaster and Humphreys (2020), our dataset was divided by considering the hardening status of plants (a binary feature; plants with and without hardening). However, there are still some plants that lack the necessary data to determine whether they have undergone hardening. Plants that have been identified as “hardened” undergo pre-acclimation through temperature treatments conducted under laboratory or greenhouse conditions. Alternatively, they may have experienced seasonal acclimation, which is documented in the original studies and highlights the seasonal variations in physiological tolerance. For species where physiological tolerance was available from multiple datasets, the average value across measurements was used. In total, our dataset summarized 882 cold tolerance records and 1,271 heat tolerance records, in 1,145 angiosperms, 99 gymnosperms, 111 ferns, and 42 bryophytes.

For each species, we retrieved occurrence data from the Global Biodiversity Information Facility (GBIF; https://www.gbif.org). We analyzed only records within the species’ native distribution areas (sensu Plants of the World Online database; https://powo.science.kew.org/). This means that any records outside the species’ original distribution areas, referred to as “outliers”, were manually excluded from the dataset. For each occurrence, we used the *extract* function in the *raster* package (version 3.5-2) to extract climatic variables of each species from 30 seconds resolution climatic data in the WorldClim 2.1 database (Fick and Hijmans, 2017). We focused on the maximum temperature of the warmest month (MTWM; Bio5) and the minimum annual temperature of the coldest month (MTCM; Bio6) for each occurrence. These two variables, MTWM and MTCM are, in fact, expected to represent the warmest and coldest temperatures in certain coordinates, potentially informing both the higher and lower bounds of climatic niche across the species range. Therefore, we described cold and warm climatic niches using 1) the average value of the MTWM and MTCM, 2) the top 90% values of MTWM (upper MTWM) and the bottom 10% of MTCM (lower MTCM) values across species’ ranges, and 3) the local MTCM or MTWM of the experimental site. As the comparison results using the upper MTWM or lower MTCM or local climate (MTCM or MTWM) were similar to those using average values (**Tables S3**, **S4**), we only reported the results derived from average values.

### 2.2 Time-calibrated phylogeny reconstruction

We used the *taxonstand* R package to standardize the species names in the database (Cayuela et al., 2012). The phylogenetic tree analyzed in this study was generated by subsampling the GBOTB.extend megatree (Smith and Brown, 2018; Jin and Qian, 2019) using the *drop.tip* function implemented in the *ape* package (Revell, 2012). The GBOTB.extend tree is the updated version of the fossil-calibrated time-tree GBOTB (Smith and Brown, 2018; Jin and Qian, 2019). This phylogeny samples over 70,000 vascular plant taxa and is widely used in macroecology and macroevolution (Landis et al., 2018; Igea and Tanentzap, 2020; Carta et al., 2022). This tree provides access to a high-resolution phylogeny of thousands of vascular plant species. From the 1,397 species with trait-level data, 42 species were not matched with the sampling in the tree. Therefore, we analyzed a total of 1,355 species with trait-level and phylogenetic information.

### 2.3 Data analyses

We compared the overall evolutionary rates of physiological tolerance to extreme temperatures and corresponding climatic-niche temperatures. Note that Lancaster and Humphreys (2020) also calculated the evolutionary rates of physiological cold and heat tolerance. However, they did not make paired comparisons between physiological tolerance and climatic niche. Here, we compared the rates between i) physiological cold tolerance (T_min_) and minimum temperatures in the coldest season (MTCM) and ii) physiological heat tolerance (T_max_) and maximum temperatures in the hottest season (MTWM). Additionally, we compared rates between iii) T_min_ and T_max_ or iv) MTCM and MTWM. We estimated the overall evolutionary rate (σ^2^) based on the Brownian motion model under maximum likelihood (Adams, 2013). We performed likelihood-ratio tests (LRTs) to test for rate differences in each group comparison. LRT values provide support to identify whether the rates are statistically different. We note that given the limited taxonomic sampling, the estimated overall rates (σ^2^) may be biased, but we only used the values in paired comparison for each comparison group. The R code associated with these analyses is presented in the supplement to this article and follows the structure of two previous studies (Adams, 2013; Liu et al., 2020b).

We also estimated absolute evolutionary rates of physiological tolerance (T_min_ and T_max_) and climatic niche (MTWM and MTCM). We estimated these rates for the i–iv sections presented above to obtain the rate comparison results between physiological tolerance and climatic niche. We calculated absolute evolutionary rates as the difference between the estimated ancestor’s state and the descendant species’ state divided by the absolute divergence time (see below for ancestral state reconstructions; Quintero and Wiens, 2013). The resulting estimate represents how much the trait changes per unit of evolutionary time for a certain species (Quintero and Wiens, 2013; Jezkova and Wiens, 2016). We used four alternative models of continuous trait evolution to reconstruct the ancestor’s state of physiological tolerance and corresponding climatic niche: Brownian motion (BM; lambda = 1) (Felsenstein, 1973), Ornstein–Uhlenbeck (OU; with one optimum value) (Butler and King, 2004), white noise (WN), and Lambda (LA; 0 < lambda < 1) (Prokoph et al., 2008). We fit the models using the *fitContinuous* function in the *geiger* R package (version 2.0.10) (Pennell et al., 2014). For each trait, we based ancestral states on the best-fitting model, which was selected for each trait as the one with the lowest Akaike information criterion (AIC). We transformed the branch lengths based on the best-fitting model, estimated the ancestor’s value, and then calculated the absolute evolutionary rates for each terminal tip. We performed paired *t*-tests to compare the absolute evolutionary rate (ln-transformed) at the species level in each group (i to iv). The results of the absolute rate comparison were similar to those of overall rates (**Tables S5**, **S6**). Finally, to test the relationship between physiological tolerance and climatic niche, we used phylogenetic generalized least squares regression (PGLS) in R package *caper* (version 1.0.1) (Orme, 2018). We performed all analyses, and we generated graphs in R 4.0.2 (R Core Team, 2020).

### 2.4 Methodological caveats

First, taxon sampling and physiological data were limited in each plant group. Because the limited sampling ratio would overestimate the branch length and thus influence the values of the calculated evolutionary rates, instead of utilizing the absolute rate value (which is with biological significance) for quantitative description, we used the overall rates and only used pairwise comparison in the same group for qualitative comparison. In this case, incomplete sampling contributed the same effect for the rate bias and did not distort the results of our comparison. Previous studies also suggested that incomplete sampling has no effect on rate comparison (Liu et al., 2020b; Qu and Wiens, 2020). Second, physiological tolerance was not discriminated against on the basis of the measuring methods as suggested by Lancaster and Humphreys (Lancaster and Humphreys, 2020; Lancaster and Humphreys, 2021). Different methods used for physiological tolerance measurements have different biological significance and values and consequently influence the rate estimation. However, because the results of the present study depended on the rate comparison, and not on rate values, the different physiological methods may have no effect on the overall tendency of the results (**Table S2**).

## 3 Results

**Figure 2:**
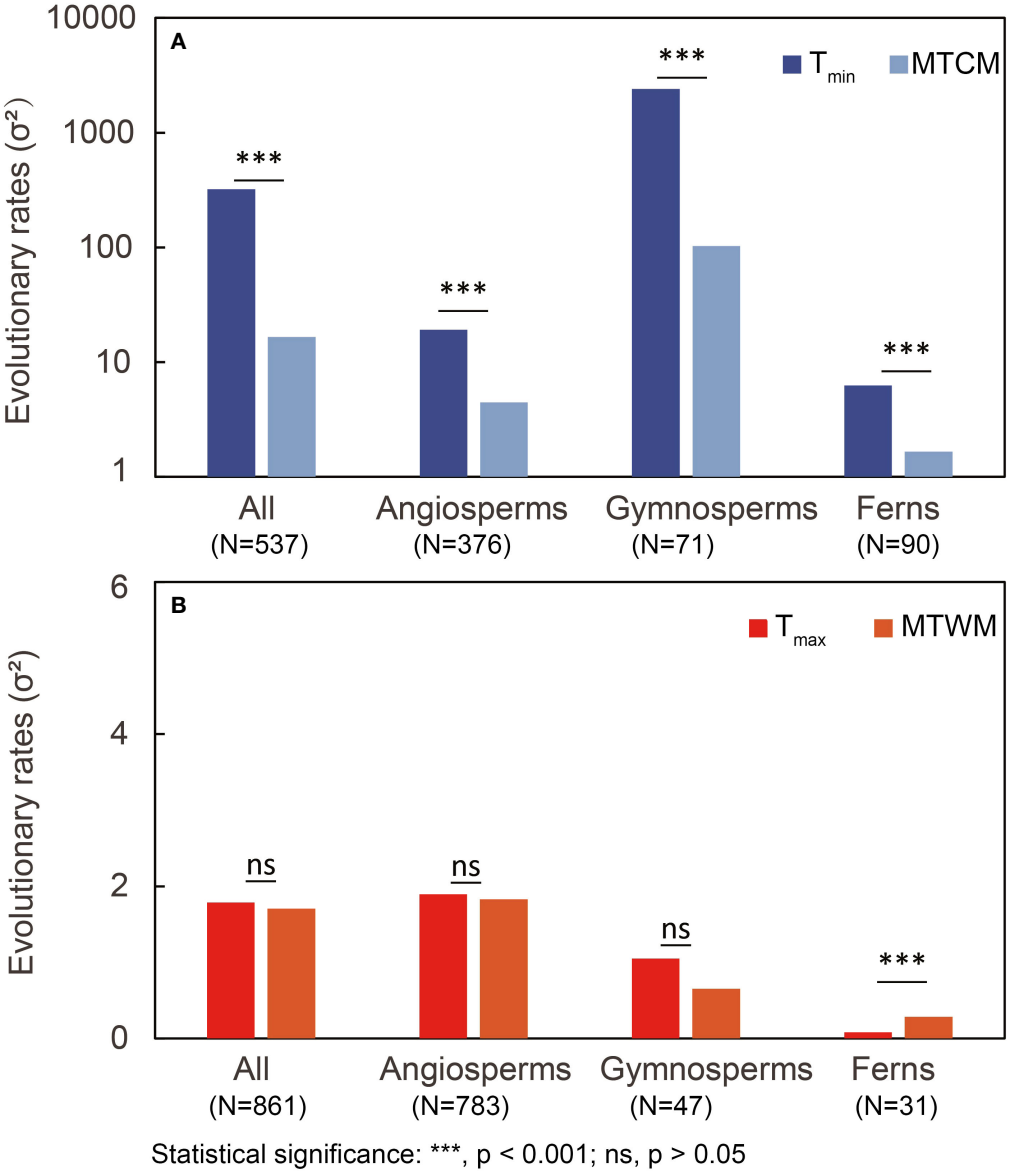
Comparison of estimated overall evolutionary rates (σ^2^) between **(A)** cold tolerance (T_min_ and minimum temperature of coldest month (MTCM)) and **(B)** heat tolerance (T_max_ and maximum temperature of warmest month (MTWM)). LRTs were conducted to obtain the p-value. N, number of species; LRT, likelihood-ratio test.

The overall evolutionary rate (σ^2^) of physiological cold tolerance (T_min_) was significantly higher than the corresponding climatic niche (MTCM) in all three major plant groups (all p < 0.001; **Figure 2**). Plants with hardening showed similar results, except the ferns that did not show significantly higher evolutionary rates in T_min_ relative to those in MTCM (**Table 1**). However, for plants without hardening, the evolutionary rates of T_min_ were lower than MTCM in all three major groups (**Table 1**).

**Table 1:** Comparison of estimated evolutionary rates (σ^2^) between physiological tolerance [cold tolerance (T_min_) and (T_max_)] and corresponding climatic niche [minimum temperature of coldest month (MTCM) and maximum temperature of warmest month (MTWM)].

Group	Type	N	Evolutionary rate (σ^2^)	LRT	p
T_min_ vs MTCM	All	537	**332.22** vs 16.55	1356.45	**<0.001**
	Hardening	264	**648.01** vs 28.62	722.86	**<0.001**
	No hardening	86	1.28 vs **2.78**	16.15	**<0.001**
Angiosperms	All	376	**19.77** vs 3.82	231.86	**<0.001**
	Hardening	201	**11.34** vs 1.79	245.25	**<0.001**
	No hardening	75	1.46 vs **2.97**	11.96	**<0.001**
	No info	169	1.92 vs **2.68**	5.29	**0.0215**
Gymnosperms	All	71	**2,399.94** vs 102.88	131.16	**<0.001**
	Hardening	56	**3,014.19** vs 128.46	103.75	**<0.001**
	no harden	5	0.0069 vs **3.22**	26.52	**<0.001**
	No info	15	**6.71** vs 2.22	13.30	**<0.001**
Ferns	All	90	**6.35** vs 1.64	56.86	**<0.001**
	Hardening	7	0.075 vs 0.12	0.58	0.4451
	No hardening	6	0.0097 vs **0.062**	5.83	**0.0157**
	No info	86	**6.60** vs 1.70	54.44	**<0.001**
T_max_ vs MTWM	All	861	1.78 vs 1.71	0.50	0.4807
	Hardening	483	1.14 vs 1.14	0.00	0.9851
	No hardening	91	0.61 vs **0.95**	4.44	**0.0351**
Angiosperms	All	783	1.90 vs 1.83	0.32	0.5687
	Hardening	442	1.18 vs 1.20	0.03	0.8687
	No hardening	87	0.60 vs **0.98**	5.38	**0.0204**
	No info	366	0.97 vs **2.21**	62.47	**<0.001**
Gymnosperms	All	47	1.05 vs 0.65	3.06	0.0801
	Hardening	36	0.76 vs 0.57	1.20	0.2726
	No hardening	4	0.82 vs 0.14	3.45	0.0633
	No info	10	0.17 vs 0.10	0.76	0.3821
Ferns	All	31	0.082 vs **0.28**	11.68	**<0.001**
	Hardening	5	0.020 vs 0.063	1.64	0.2002
	No hardening	–	–	–	–
	No info	26	0.084 vs **0.25**	7.40	**0.0065**

Bold font indicates the higher evolutionary rates. LRTs were conducted to obtain the p-value. “-” indicate no data.

N, number of species; LRT, likelihood-ratio test.

In terms of heat tolerance for gymnosperms and angiosperms, there were no significant differences between the rates of physiological tolerance (T_max_) and corresponding climatic niche (MTWM). In ferns, T_max_ evolved slower than MTWM (**Figure 2**). Plants with hardening showed no significant difference in the evolutionary rates of T_max_ and MTWM (**Table 1**).

**Figure 3:**
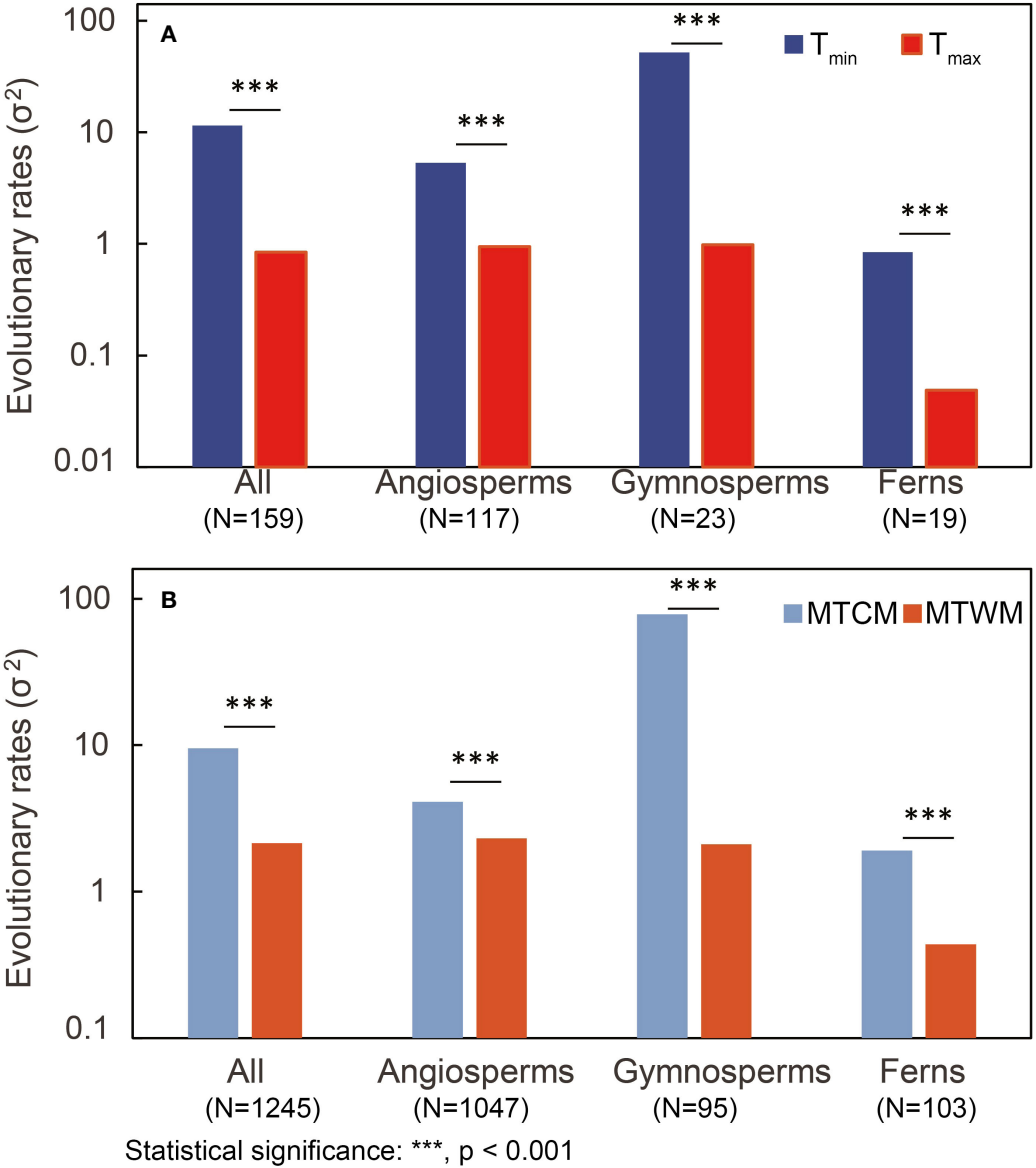
Comparison of estimated evolutionary rates (σ^2^) between physiological tolerance and responding climatic niche in both **(A)** cold tolerance [minimum temperature of coldest month (MTCM) versus physiological tolerance (T_min_)] and **(B)** heat tolerance [maximum temperature of warmest month (MTWM) versus physiological tolerance (T_max_)] among different plant groups. LRTs were conducted to obtain the p-value. N, number of species; LRT, likelihood-ratio test.

**Figure 4:**
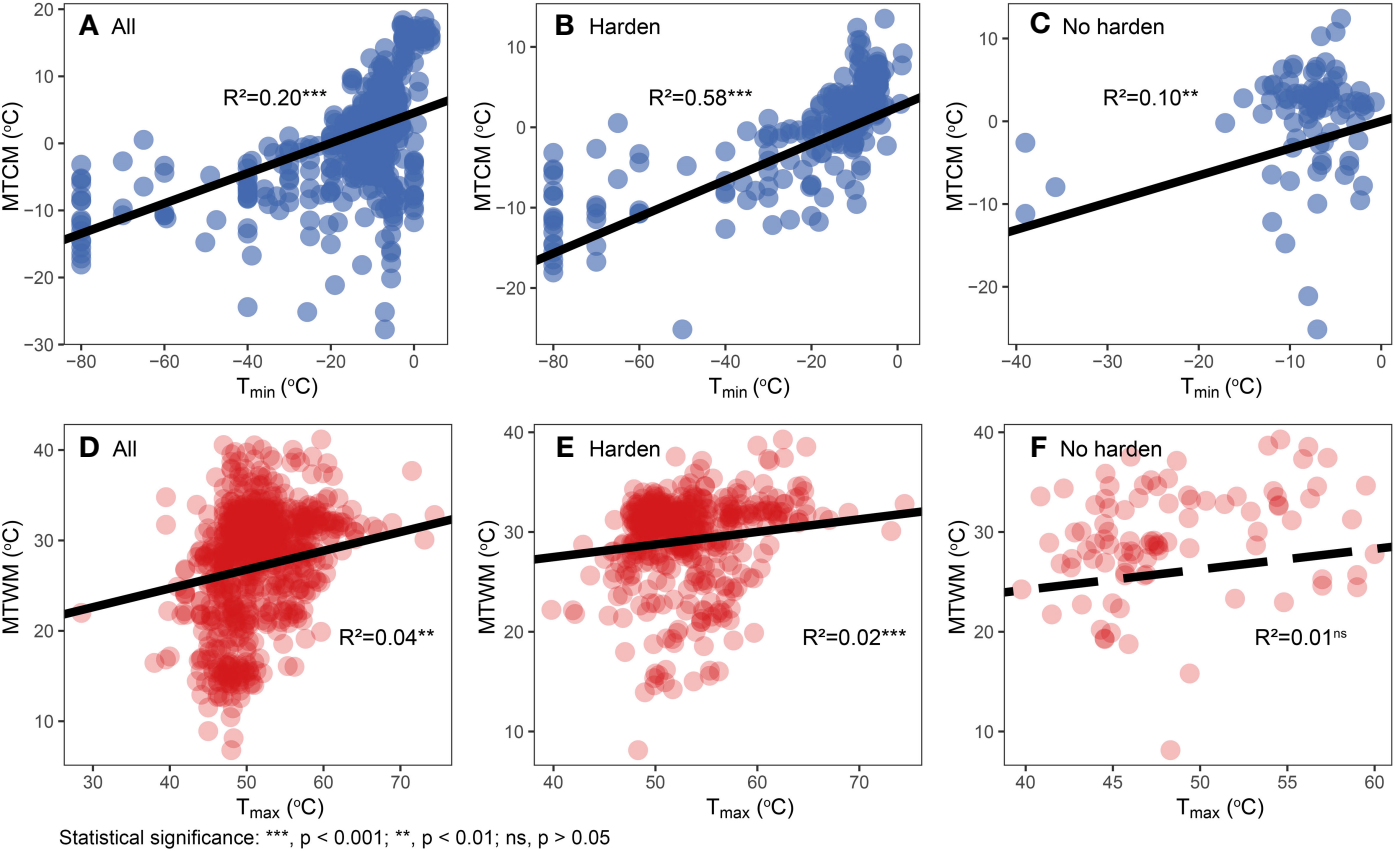
Relationships between physiological cold tolerance and corresponding climatic niche. **(A–C)** Physiological cold tolerance (T_min_) versus minimum temperature of coldest month (MTCM). **(D–F)** Physiological heat tolerance (T_max_) versus maximum temperature of warmest month (MTWM). Different hardening statuses were presented in different plots: all data **(A, D)**, hardened **(B, E)**, and not hardened **(C, F)**. Regression lines, R^2^, and p-values are based on phylogenetic generalized least squares (PGLS) regression analyses.

We found cold tolerance, in both physiological and climatic niches, to evolve faster than corresponding heat tolerance in all three major groups (p < 0.001) (**Figure 3**; **Table S7**). T_min_ was significantly correlated with MTCM (R^2 = ^0.20, p < 0.001), whereas the correlation of hardened plants was stronger (R^2 = ^0.58, p < 0.001) than that of non-hardened plants (R^2 = ^0.10, p < 0.001). For T_max_, a significant but weak correlation between T_max_ and MTWM was found in all species (R^2 = ^0.04, p < 0.001) and hardened plants (R^2 = ^0.02, p < 0.001), but not in non-hardened plants (R^2 = ^0.01, p = 0.12) (**Figure 4**).

## 4 Discussion

We leveraged a large species-level dataset on species climatic responses based on physiological and climatic niches, combined with phylogenetic information. We found that the evolutionary rates of physiological cold tolerance were higher than those of the corresponding climatic niche, while the cold tolerance of plants without hardening showed the opposite trend. However, the evolutionary rates of physiological heat tolerance were not different from those of the corresponding climatic niche, in both hardening and non-hardening plants. Our results suggest that physiological responses are likely the basis of ecological adaptation under cold stress. Consistent with previous studies (Lancaster and Humphreys, 2020; Liu et al., 2020b), we show that cold tolerance evolves faster than heat tolerance in both physiological tolerance and climatic niche. Thus, cold temperatures may be one of the key drivers of plant evolution. However, the limited evolutionary rate of heat tolerance may limit the future performance of plants in the coming warm world.

### 4.1 Physiological tolerances underlie climatic niches

Our results show that physiological cold tolerance evolved faster than the responding climatic niche for hardened plants, whereas the non-hardened plants showed the opposite trend. First, local microclimate, rather than macroclimate, could explain the plant responses in communities because it is directly the temperature that plant individuals experience and is more diverse compared to sole and more stable macroclimate (Zellweger et al., 2020). Physiological tolerance faces a higher selective pressure for the sensitivity of the physiological process to microclimate change, therefore showing higher evolutionary rates than macro climatic niches. Second, hardened plants in physiological cold tolerance and physiological heat tolerance were not consistent with the higher rates in climatic niches reported for animals (Qu and Wiens, 2020). This may be attributed to the ability of animals to move in order to avoid extreme temperatures (behavior avoidance) or to keep body temperature largely constant (homeothermy) (Hetem et al., 2012; Pereira et al., 2020). In contrast, plant migration is achieved only in next-generation individuals via seed dispersal, which is a slow process that lags behind climate warming (Corlett and Westcott, 2013). Therefore, physiological changes or acclimation are essential for the survival of plants.

Our results highlight the importance of hardening to physiological tolerance, especially to cold tolerance. Previous studies have addressed the physiological basis of the hardening effect on plant tolerance; for example, the changes in carbohydrate metabolism (Knaupp et al., 2011) and downregulation of photosynthesis (Frechette et al., 2015) enhanced cold tolerance in a relatively short time. This hardening process also accelerates the evolutionary rate of physiological tolerance at higher rates than the responding climatic niche. However, without hardening, plants with limited tolerance to both cold and heat contributed to lower or similar evolutionary rates than the corresponding climatic niche. In this case, the results were partly consistent with the observations in animals (Qu and Wiens, 2020).

It has been suggested that the physiological tolerance to extreme temperatures is correlated to the species’ climatic niche. However, our study revealed enhanced correlations in hardened plants and weakened relationships in non-hardened plants, suggesting that these correlations may partly be due to the physiological acclimation to natural climate-induced hardening. Thus, our results indicate that physiological tolerance, especially cold tolerance, constituted the basis of the climatic niche.

### 4.2 Why does cold tolerance evolve faster than heat tolerance?

Several potential explanations have been postulated regarding the higher rates of cold tolerance. First, the spatial distribution of the Earth’s surface temperature shapes both physiological tolerance and climatic niche. The lower variance in annual maximum temperature relative to minimum temperature across Earth biomes contributes to lower variance of heat tolerance and consequently lowers the rates of evolution for heat tolerance. Second, the historical thermal dynamics in geological time could have influenced plant evolution. For instance, at ~65 Ma, the surface temperature of Earth became cooler, despite a few rapid warming events (e.g., PETM and the last glacial termination) (Prokoph et al., 2008; Veizer and Prokoph, 2015). Cold climates were thus the main drivers of plant adaptation to the environment because high temperatures were relatively absent. These also contributed to the strong correlations between physiological cold tolerance and MTCM and the weak correlations between heat tolerance and MTWM. Third, the ancestors of most major clades of land plants (e.g., angiosperms) could have originated in warm-climate areas, but the descendants migrated and radiated into cooler areas; therefore, cold tolerance evolution is needed. One classic example is the poleward expansion of angiosperms during the Cenozoic (Zanne et al., 2014). Fourth, the evolution of climatic adaptation might also be associated with the macroevolution and diversification of plants. The younger age and higher speciation rates of plant taxa in cooler climates and higher latitudes indicate their potential higher selective pressure in cool areas (Lu et al., 2018; Igea and Tanentzap, 2020), further suggesting the cold climates may be the main driver in the macroevolution of plants. Some of the ideas are not exclusive of each other; however, they suggest alternative but potentially overlapping paths explaining faster cold tolerance evolution in plants.

### 4.3 Plants may be more sensitive to projected warming but still affected by cold hazards

Lower evolutionary rates of heat tolerance, as well as its narrow variance (Araujo et al., 2013; Lancaster and Humphreys, 2020), indicate that plants may have difficulty adapting to warmer climates. Our results also revealed that even for hardened plants, the rates of heat tolerance were not significantly higher than MTWM, indicating the limited plasticity of heat tolerance and higher risk of plants to climate warming. Particularly for tropical plants, existence in a uniformly warm climate after the last glacial maximum is conservative to heat tolerance and with increased risk of climate warming (Costion et al., 2015; Perez and Feeley, 2020; Tierney et al., 2020).

The rates of current warming are considerably higher than they had been at any geological time in the past, whereas the evolutionary rates of climatic niches are lower than those of climate change (Quintero and Wiens, 2013; Jezkova and Wiens, 2016). As the current global mean temperature is near the maximum value estimated for the past 1.2 million years (Foster et al., 2017; Steffen et al., 2018), such higher temperatures and warming are unnatural and may impact the global ecosystem similar to the past rapid warming events in geological history (Jaramillo et al., 2010; Bellard et al., 2012; Wing and Currano, 2013; Smith et al., 2020). In addition to heat, the cold hazards may affect plants, especially populations of recently latitudinally or altitudinally migrated plants, which would experience more frequent cold hazards than native habitats. Cold events are projected to occur with high anomaly (e.g., occurrence of frost events in spring after unusual warming) (Ipcc, 2014; Vitasse et al., 2018). Cold hazard affects the physiological performance of plants and slows down their migration to cooler climate areas (Wen et al., 2018). Therefore, it is necessary to assess the physiological tolerance to heat and cold tolerance of plants for their future conservation and potential risk to climate change.

## 5 Conclusions

The evolutionary rates of physiological cold tolerance were higher than those of the corresponding climatic niche, while the cold tolerance without hardening showed the opposite trend, suggesting that physiological tolerance constitutes the basis of the climatic niche. Physiological heat tolerance was not evolving faster than the corresponding climatic niche in both hardening and non-hardening plants. Cold tolerance evolved faster than heat tolerance in both physiological tolerance and climatic niche. Plants may be sensitive and with a high risk of future warming, and assessing the physiological tolerance of plants is a prerequisite for their successful conservation.

## Data availability statement

The datasets presented in this study can be found in online repositories. The names of the repository/repositories and accession number(s) can be found below: https://figshare.com/s/06708b691f153febca98.

## Author contributions

YW: Conceptualization, Writing – review & editing, Formal Analysis, Visualization, Writing – original draft. QY: Conceptualization, Writing – review & editing, Supervision. CR: Writing – review & editing. HL: Writing – review & editing. GW: Writing – review & editing.
