# Supplementary material for: Physiological cold tolerance evolves faster than climatic niches in plants
Source: Front Plant Sci. 2023 Sep 8;14:1257499. doi: 10.3389/fpls.2023.1257499 (PMC10515087; doi:10.3389/fpls.2023.1257499)
Supplement: Supplementary file 1 [file DataSheet_1.docx]

Supplementary Material

Physiological cold tolerance evolves faster than climatic niches in plants

Yin Wen^1^, Qing Ye^1,2*^, Cristian Román-Palacios^3^, Hui Liu^1^, Gui-Lin Wu^1^

^1^Key Laboratory of Vegetation Restoration and Management of Degraded Ecosystems, Guangdong Provincial Key Laboratory of Applied Botany, South China Botanical Garden, Chinese Academy of Sciences, Xingke Road 723, Guangzhou, 510650, China

^2^ College of Life Sciences, Gannan Normal University, Ganzhou, 341000, China

^3^ School of Information, University of Arizona, Tucson, Arizona, 85721, USA

*** Correspondence:** Qing Ye: qye@scbg.ac.cn

**Table S1** Data references.

| Alberdi, M., Meza-Basso, L., Fernandez, J., Rios, D. & Romero, M. 1989. Seasonal changes in carbohydrate content and frost resistance of leaves of Nothofagus species. *Phytochemistry* **28**: 759–763. |
| --- |
| Armstrong, J.J., Takebayashi, N., Sformo, T. & Wolf, D.E. 2015. Cold tolerance in *Arabidopsis kamchatica*. *Am. J. Bot.* **102**: 439–448. John Wiley & Sons, Ltd.  Balagurova, N., Drozdov, S. & Grabovik, S.1996. Cold and heat resistance of five species of Sphagnum. *Annales Botanici Fennici*. **33**, 33-37. |
| Bannister, P. 2007. Godley review: A touch of frost? Cold hardiness of plants in the southern hemisphere. *New Zeal. J. Bot.* **45**: 1–33.  Bannister, P. & Lord, J. M. 2006. Comparative winter frost resistance of plant species from southern Africa, Australia, New Zealand, and South America grown in a common environment (Dunedin, New Zealand). *New Zealand Journal of Botany* **44**, 109-119. |
| Bannister, P., Maegli, T., Dickinson, K.J.M., Halloy, S.R.P., Knight, A., Lord, J.M., *et al.* 2005. Will loss of snow cover during climatic warming expose New Zealand alpine plants to increased frost damage? *Oecologia* **144**: 245–256. |
| Bannister, P. & Polwart, A. 2001. *The Frost Resistance of Ericoid Heath Plants in the British Isles in Relation to Their Biogeography*. |
| Bauer, A. & Black, A.L. 1990. Stubble Height Effect on Winter Wheat in the Northern Great Plains: I. Soil Temperature, Cold Degree-Hours, and Plant Population. *Agron. J.* **82**: 195. American Society of Agronomy. |
| Beck, E. 1994. Cold tolerance in tropical alpine plants. In: *Tropical alpine environments : plant form and function* (P. W. (Philip W. Rundel et al., eds), pp. 77–110. Cambridge University Press. |
| Berry, J. & Bjorkman, O. 1980. Photosynthetic Response and Adaptation to Temperature in Higher Plants. *Annu. Rev. Plant Physiol.* **31**: 491–543. |
| Biebl, R. 1964. Temperaturresistenz tropischer Pflanzen auf Puerto Rico. *Protoplasma* **59**: 133–156. |
| Biebl, R. 1967. Über Wärmehaushalt und Temperaturresistenz arktischer Pflanzen in Westgrönland. *Flora oder Allg. Bot. Zeitung. Abt. B, Morphol. und Geobot.* **157**: 327–354.  Bilger, H.-W., Schreiber, U. & Lange, O. 1984. Determination of leaf heat resistance: comparative investigation of chlorophyll fluorescence changes and tissue necrosis methods. *Oecologia* **63**, 256-262. |
| Blake, J. & Hill, R.S. 1996. An examination of the drought and frost tolerance of Banksia marginata (proteaceae) as an explanation of its current widespread occurrence in Tasmania. *Aust. J. Bot.* **44**: 265–281. |
| Braun, V., Buchner, O. & Neuner, G. 2002. Thermotolerance of Photosystem 2 of Three Alpine Plant Species Under Field Conditions. *Photosynthetica* **40**: 587–595. |
| Bravo, L.A., Ulloa, N., Zuñiga, G.E., Casanova, A., Corcuera, L.J. & Alberdi, M. 2001. Cold resistance in antarctic angiosperms. *Physiol. Plant.* **111**: 55–65.  Buchner, O. & Neuner, G.2010. Freezing cytorrhysis and critical temperature thresholds for photosystem II in the peat moss Sphagnum capillifolium. *Protoplasma* **243**, 63-71. |
| Buchner, O., Stoll, M., Karadar, M., Kranner, I. & Neuner, G. 2015. Application of heat stress in situ demonstrates a protective role of irradiation on photosynthetic performance in alpine plants. *Plant. Cell Environ.* **38**: 812–26.  Bykova, O. & Sage, R. F.2012. Winter cold tolerance and the geographic range separation of Bromus tectorum and Bromus rubens, two severe invasive species in North America. *Glob. Change Biol*. **18**, 3654-3663.  Cieraad, E., McGlone, M., Barbour, M. M. & Huntley, B.2012. Seasonal Frost Tolerance of Trees in the New Zealand Treeline Ecotone. *Arctic, Antarctic, and Alpine Research* **44**, 332-342. |
| Charra-Vaskou, K., Charrier, G., Wortemann, R., Beikircher, B., Cochard, H., Ameglio, T., *et al.* 2012. Drought and frost resistance of trees: a comparison of four species at different sites and altitudes. *Ann. For. Sci.* **69**: 325–333. |
| Chaves, C.J.N., Leal, B.S.S. & Lemos-Filho, J.P. de. 2018. How are endemic and widely distributed bromeliads responding to warming temperatures? A case study in a Brazilian hotspot. *Flora* **238**: 110–118. |
| Cunningham, S.C. & Read, J. 2006. Foliar temperature tolerance of temperate and tropical evergreen rain forest trees of Australia. *Tree Physiol.* **26**: 1435–1443. |
| Curtis, E.M. 2017. Spatiotemporal dynamics of high-temperature tolerance in Australian arid-zone plants. |
| Darrow, H.E., Bannister, P., Burritt, D.J. & Jameson, P.E. 2001. The frost resistance of juvenile and adult forms of some heteroblastic New Zealand plants. *New Zeal. J. Bot.* **39**: 355–363. |
| Fiorino, P. & Mancuso, S. 2000. Differential thermal analysis, supercooling and cell viability in organs of Olea europaea at subzero temperatures. Advances in Horticultural Science. **14**: 23-27.  Froux, F., Ducrey, M., Epron, D. & Dreyer, E. 2004. Seasonal variations and acclimation potential of the thermostability of photochemistry in four Mediterranean conifers. *Annals of Forest Science* **61**, 235-241. |
| Garrett, T.Y., Huynh, C.-V. & North, G.B. 2010. Root contraction helps protect the “living rock” cactus *Ariocarpus fissuratus* from lethal high temperatures when growing in rocky soil. *Am. J. Bot.* **97**: 1951–1960. |
| Gauslaa, Y. 1984. *Heat Resistance and Energy Budget in Different Scandinavian Plants*.  Ghouil, H. et al. 2003. Thermal optima of photosynthetic functions and thermostability of photochemistry in cork oak seedlings. *Tree Physiology* **23**, 1031-1039 (2003). |
| Gianoli, E., Gianoli, E., Inostroza, P., Zúñiga-Feest, A., Zúñiga-Feest, A., Reyes-Díaz, M., *et al.* 2004. Ecotypic Differentiation in Morphology and Cold Resistance in Populations of Colobanthus quitensis (Caryophyllaceae) from the Andes of Central Chile and the Maritime Antarctic **36**: 484–489.  Gimeno, T. E., Pías, B., Lemos-Filho, J. P. & Valladares, F. 2009. Plasticity and stress tolerance override local adaptation in the responses of Mediterranean holm oak seedlings to drought and cold. *Tree Physiology* **29**, 87-98.  Godoy, O., de Lemos-Filho, J. P. & Valladares, F. 2011. Invasive species can handle higher leaf temperature under water stress than Mediterranean natives. *Environmental and Experimental Botany* **71**, 207-214. |
| Goldstein, G., Rada, F. & Azocar, A. 1985. Cold hardiness and supercooling along an altitudinal gradient in andean giant rosette species. *Oecologia* **68**: 147–152. Springer-Verlag. |
| Guo, H., Gao, S., Zhao, F. & Li, F. 2004. Effects of cold acclimation on several enzyme activities in Euonymus radicans “emorald &amp; gold” and its relation to semi-lethal temperature. *For. Stud. China* **6**: 10–17.  Gurney, K. M., Schaberg, P. G., Hawley, G. J. & Shane, J. B. 2011. Inadequate Cold Tolerance as a Possible Limitation to American Chestnut Restoration in the Northeastern United States. *Restoration Ecology* **19**, 55-63. |
| Jacobsen, S.-E., Monteros, C., Corcuera, L.J., Bravo, L.A., Christiansen, J.L. & Mujica, A. 2007. Frost resistance mechanisms in quinoa (Chenopodium quinoa Willd.). *Eur. J. Agron.* **26**: 471–475. |
| Kappen, L. 1964. Untersuchungen über den Jahreslauf der Frost-, Hitze- und Austrocknungsresistenz von Sporophyten einheimischer Polypodiaceen (Filicinae). *Flora oder Allg. Bot. Zeitung* **155**: 123–166. |
| Knight, C.A. & Ackerly, D.D. 2002. An ecological and evolutionary analysis of photosynthetic thermotolerance using the temperature-dependent increase in fluorescence. *Oecologia* **130**: 505–514. |
| Konis, E. 1949. *The Resistance of Maquis Plants to Supramaximal Temperatures*. |
| Krause, G.H., Winter, K., Krause, B., Jahns, P., García, M., Aranda, J., *et al.* 2010. High-temperature tolerance of a tropical tree, Ficus insipida: Methodological reassessment and climate change considerations. *Funct. Plant Biol.* **37**: 890–900. |
| Lange, O.L. 1959. Untersuchungen über Wärmehaushalt und Hitzeresistenz mauretanischer Wüsten- und Savannenpflanzen. *Flora oder Allg. Bot. Zeitung* **147**: 595–651. |
| Lange, O.L. & Lange, R. 1962. Die Hitzeresistenz einiger mediterraner Pflanzen in Abhängigkeit von der Höhenlage ihrer Standorte. *Flora* **152**: 707–710. |
| Lange, O.L. & Lange, R. 1963. Untersuchungen über Blattemperaturen, Transpiration und Hitzeresistenz an Pflanzen mediterraner Standorte (Costa brava, Spanien). *Flora oder Allg. Bot. Zeitung* **153**: 387–425. |
| Larcher, W. 1954. Die Kälteresistenz Mediterraner immergrüner und ihre beeinflussbarkeit. *Planta* **44**: 607–635. |
| Larcher, W. 2000. Temperature stress and survival ability of Mediterranean sclerophyllous plants. *Plant Biosyst.* **134**. |
| Larcher, W., Holzner, M. & Pichler, J. 1989. Temperaturresistenz inneralpiner Trockenrasen / Temperature Resistance of Graminoids from a Dry Valley of the Central Alps. *Flora* **183**: 115–131. |
| Larcher, W. & Wagner, J. 1976. Temperaturgrenzen der CO2- Aufnahme und Temperaturresistenz der Blätter von Gebirgspflanzen im vegetationsaktiven Zustand. *Oecol Plant* **11**: 361–374. |
| Lipp, C.C., Goldstein, G., Meinzer, F.C. & Niemczura, W. 1994. Freezing tolerance and avoidance in high-elevation Hawaiian plants. *Plant, Cell Environ.* **17**: 1035–1044.  Liu, Y., Cao, T. & Glime, J. M. 2003. The Changes of Membrane Permeability of Mosses under High Temperature Stress. *The Bryologist* **106**, 53-60. |
| Loik, M.E. & Harte, J. 1996. *International Association for Ecology High-Temperature Tolerance of Artemisia tridentata and Potentilla gracilis under a Climate Change Manipulation*. |
| Loik, M.E. & Nobel, P.S. 1993. *Freezing Tolerance and Water Relations of Opuntia Fragilis from Canada and the United*. |
| Lösch, R. 1980. Die Hitzeresistenz der Pflanzen des kanarischen Lorbeerwaldes. *Flora* **170**: 456–465. |
| Maier, R. 1971. Einfluß von Photoperiode und Einstrahlungsstärke auf die Temperaturresistenz einiger Samenpflanzen. *Österreichische Bot. Zeitschrift* **119**: 306–322. |
| Marias, D.E., Meinzer, F.C., Woodruff, D.R. & McCulloh, K.A. 2016. Thermotolerance and heat stress responses of Douglas-fir and ponderosa pine seedling populations from contrasting climates. *Tree Physiol.* **37**: 301–315.  Meyer, H. & Santarius, K. A. 1998. Short-term thermal acclimation and heat tolerance of gametophytes of mosses. *Oecologia* **115**, 1-8. |
| Murphy, B. & Way, D. 2017. Future climate conditions alter leaf thermotolerance in Canadian boreal trees. Undergraduate Thesis. Western University, Canada.  Neuner, G., Buchner, O. & Braun, V. 2000. Short-Term Changes in Heat Tolerance in the Alpine Cushion Plant Silene acaulis ssp. excapa [All.] J. Braun at Different Altitudes. *Plant Biology* **2**, 677-683. |
| Nobel, P.S. 1984. Extreme temperatures and thermal tolerances for seedlings of desert succulents. *Oecologia* **62**: 310–317. |
| Nobel, P.S. 1982. Low-Temperature Tolerance and Cold Hardening of Cacti. *Ecology* **63**: 1650. |
| Nobel, P.S. & Smith, S.D. 1983. High and low temperature tolerances and their relationships to distribution of agaves. *Plant, Cell Environ.* **6**: 711–719. |
| O’sullivan, O.S., Heskel, M.A., Reich, P.B., Tjoelker, M.G., Weerasinghe, L.K., Penillard, A., *et al.* 2017. Thermal limits of leaf metabolism across biomes. *Glob. Chang. Biol.* **23**: 209–223. |
| O’Sullivan, O.S., Weerasinghe, K.W.L.K., Evans, J.R., Egerton, J.J.G., Tjoelker, M.G. & Atkin, O.K. 2013. High-resolution temperature responses of leaf respiration in snow gum (Eucalyptus pauciflora) reveal high-temperature limits to respiratory function. *Plant, Cell Environ.* **36**: 1268–1284. |
| Offord, C.A. 2011. Pushed to the limit: consequences of climate change for the Araucariaceae: a relictual rain forest family. *Ann. Bot.* **108**: 347–357. |
| Ortiz, C. & Cardemil, L. 2001. Heat-shock responses in two leguminous plants: a comparative study. *J. Exp. Bot.* **52**: 1711–1719. |
| Patiño, S. & Grace, J. 2002. The cooling of convolvulaceous flowers in a tropical environment. *Plant. Cell Environ.* **25**: 41–51.  Pérez, F., Hinojosa, L. F., Ossa, C. G., Campano, F. & Orrego, F. 2014. Decoupled evolution of foliar freezing resistance, temperature niche and morphological leaf traits in Chilean Myrceugenia. *Journal of Ecology* **102**, 972-980.  Perez, TM, Feeley, KJ. 2020. Weak phylogenetic and climatic signals in plant heat tolerance. *J. Biogeogr*. **00**: 1– 10. |
| Pompeiano, A., Vita, F., Miele, S. & Guglielminetti, L. 2015. Freeze tolerance and physiological changes during cold acclimation of giant reed [ *Arundo donax* (L.)]. *Grass Forage Sci.* **70**: 168–175. |
| Read, J. & Hill, R.S. 1989. The Response of Some Australian Temperate Rain Forest Tree Species to Freezing Temperatures and Its Biogeographical Significance. *J. Biogeogr.* **16**: 21–27. |
| Reyes, M.A., Corcuera, L.J. & Cardemil, L. 2003. Accumulation of HSP70 in Deschampsia antarctica Desv. leaves under thermal stress. *Antarct. Sci.* **15**: 345–352.  Rütten, D. & Santarius, K. A. 1992. Relationship between Frost Tolerance and Sugar Concentration of Various Bryophytes in Summer and Winter. *Oecologia* **91**, 260-265. |
| Sakai, A. & Larcher, W. 1987. *Frost Survival of Plants : Responses and Adaptation to Freezing Stress*. |
| Sakai, A. & Ōtsuka, K. 1970. *Freezing Resistance of Alpine Plants*. |
| Sakai, A. & Wardle, P. 1978. Freezing Resistance of New Zealand Trees and Shrubs. *N. Z. J. Ecol.* **1**: 51–61. |
| Sakai, A. & Weiser, C.J. 1973. *Freezing Resistance of Trees in North America with Reference to Tree Regions*. |
| Sastry, A., Guha, A. & Barua, D. 2018. Leaf thermotolerance in dry tropical forest tree species: relationships with leaf traits and effects of drought. *AoB Plants* **10**. |
| Sato, T. 1982. Phenology and wintering capacity of sporophytes and gametophytes of ferns native to northern Japan. *Oecologia* **55**: 53–61. |
| Sklenář, P., Kučerová, A., Macek, P. & Macková, J. 2010. Does plant height determine the freezing resistance in the páramo plants? *Austral Ecol.* **35**: 929–934. |
| Slot, M., Garcia, M.N. & Winter, K. 2016. Temperature response of CO2 exchange in three tropical tree species. *Funct. Plant Biol.* **43**: 468–478. |
| Smith, S.D., Didden-Zopfy, B. & Nobel, P.S. 1984. *High-Temperature Responses of North American Cacti*.  Slot, M., Cala, D., Aranda, J., Virgo, A., Michaletz, S. T., & Winter, K. 2021. Leaf heat tolerance of 147 tropical forest species varies with elevation and leaf functional traits, but not with phylogeny. Plant Cell and Environment. |
| Squeo, F.A., Rada, F., Azocar, A. & Goldstein, G. 1991. Freezing tolerance and avoidance in high tropical Andean plants: Is it equally represented in species with different plant height? *Oecologia* **86**: 378–382. |
| Svenning, M.M., Rosnes, K. & Junttila, O. 1997. Frost tolerance and biochemical changes during hardening and dehardening in contrasting white clover populations. *Physiol. Plant.* **101**: 31–37. |
| Taschler, D., Beikircher, B. & Neuner, G. 2004. Frost resistance and ice nucleation in leaves of five woody timberline species measured in situ during shoot expansion. *Tree Physiol.* **24**: 331–337. |
| Taulavuori, E., Tahkokorpi, M., Taulavuori, K. & Laine, K. 2004. Anthocyanins and glutathione S-transferase activities in response to low temperature and frost hardening in Vaccinium myrtillus (L.). *J. Plant Physiol.* **161**: 903–911. |
| Zhang, J.-L., Poorter, L., Hao, G.-Y. & Cao, K.-F. 2012. Photosynthetic thermotolerance of woody savanna species in China is correlated with leaf life span. *Ann. Bot.* **110**: 1027–1033. |
| Zhu, L., Bloomfield, K.J., Hocart, C.H., Egerton, J.J.G., O’Sullivan, O.S., Penillard, A., *et al.* 2018. Plasticity of photosynthetic heat tolerance in plants adapted to thermally contrasting biomes. *Plant. Cell Environ.* **41**: 1251–1262. |

| Group | Type | N | Evolutionary rate (σ^2^) | LRT | p |
| --- | --- | --- | --- | --- | --- |
| T_min_ vs. MTCM |  | 537 | **332.22** vs. 16.55 | 1356.45 | **<0.001** |
| FR | all | 139 | **1230.72** vs. 54.37 | 380.54 | **<0.001** |
|  | harden | 105 | **1621.24** vs. 69.52 | 193.94 | **<0.001** |
|  | no harden | - | - | - | - |
|  | no.info | 38 | 2.82 vs. **5.46** | 10.12 | **0.0014** |
| FT | all | 13 | **1.66** vs. 0.10 | 19.47 | **<0.001** |
|  | harden | 8 | 0.070 vs. 0.12 | 0.68 | 0.4091 |
|  | no harden | 5 | 0.017 vs. **0.99** | 13.70 | **<0.001** |
|  | no.info | 5 | **4.25** vs. 0.017 | 26.80 | **<0.001** |
| LT_100_ | all | 21 | 0.13 vs. **0.72** | 18.73 | **<0.001** |
|  | harden | 12 | 0.027 vs. 0.063 | 3.20 | 0.0738 |
|  | no harden | 12 | 0.021 vs. **0.063** | 4.30 | **0.0381** |
|  | no.info | 9 | 0.25 vs. 0.55 | 2.79 | 0.0951 |
| LT_50_ | all | 380 | **1.84** vs. 1.52 | 4.52 | **0.033** |
|  | harden | 154 | 0.97 vs. **0.61** | 16.38 | **<0.001** |
|  | no harden | 63 | 1.01 vs. **3.16** | 28.04 | **<0.001** |
|  | no.info | 209 | **2.00** vs. 1.31 | 10.69 | **0.0108** |
| No data | - | 13 | **1.77** vs. 0.50 | 9.69 | **0.0019** |
| T_max_ vs. MTWM |  | 861 | 1.78 vs. 1.71 | 0.50 | 0.4807 |
| LT_100_ |  | 8 | 0.064 vs. 0.42 | 6.36 | **0.0116** |
| LT_50_ | all | 648 | 0.60 vs. **0.82** | 16.43 | **<0.001** |
|  | harden | 335 | 0.29 vs. 0.36 | 3.71 | 0.0541 |
|  | no harden | 90 | 0.59 vs. **0.94** | 5.12 | **0.0236** |
|  | no.info | 322 | 0.66 vs. **0.99** | 12.95 | **<0.001** |
| T_crit_ |  | 14 | 0.067 vs. 0.034 | 1.69 | 0.1932 |
| T_max_ | all | 269 | 0.084 vs. **0.25** | 7.40 | **0.0065** |
|  | harden | 198 | 1.35 vs. **2.05** | 15.61 | **<0.001** |
|  | no harden | - | - | - | - |
|  | no.info | 71 | 1.22 vs. **5.56** | 51.56 | **<0.001** |

**Table S2** Comparison of estimated evolutionary rates (σ^2^) between physiological tolerance (T_min_ and T_max_) and related climatic niche (MTCM and MTWM). N, number of species. LRT, likelihood-ratio test. NA, no data.

| Group | Type | N | Evolutionary rate (σ^2^) | LRT | p |
| --- | --- | --- | --- | --- | --- |
| T_min_ vs. lower MTCM | all | 537 | **332.22** vs. 54.40 | 878.52 | **<0.001** |
|  | harden | 264 | **648.01** vs. 104.06 | 450.78 | **<0.001** |
|  | no harden | 86 | 1.28 vs. **3.38** | 24.14 | **<0.001** |
| Angiosperms | all | 376 | **19.77** vs. 4.25 | 215.07 | **<0.001** |
|  | harden | 201 | **11.34** vs. 2.61 | 171.01 | **<0.001** |
|  | no harden | 75 | 1.46 vs. **3.26** | 15.16 | **<0.001** |
|  | no.info | 169 | 1.92 vs. **3.83** | 21.35 | **<0.001** |
| Gymnosperms | all | 71 | **2399.94** vs. 383.81 | 52.79 | **<0.001** |
|  | harden | 56 | **3014.19** vs. 481.16 | 41.71 | **<0.001** |
|  | no harden | 5 | 0.0069 vs. **9.17** | 31.63 | **<0.001** |
|  | no.info | 15 | 6.71 vs. 11.28 | 3.18 | 0.0747 |
| Ferns | all | 90 | **6.35** vs. 4.04 | 9.94 | **0.0016** |
|  | harden | 7 | 0.075 vs. **0.28** | 4.59 | **0.0322** |
|  | no harden | 6 | 0.0097 vs. **0.085** | 6.41 | **0.0113** |
|  | no.info | 86 | **6.60** vs. 4.21 | 9.45 | **0.0021** |
| T_max_ vs. upper MTWM | all | 861 | **1.78** vs. 1.39 | 13.49 | **<0.001** |
|  | harden | 483 | **1.14** vs. 0.77 | 21.85 | **<0.001** |
|  | no harden | 91 | 0.61 vs. 0.86 | 2.63 | 0.1048 |
| Angiosperms | all | 783 | **1.90** vs. 1.49 | 13.26 | **<0.001** |
|  | harden | 442 | **1.18** vs. 0.81 | 20.10 | **<0.001** |
|  | no harden | 87 | 0.60 vs. 0.89 | 3.27 | 0.0705 |
|  | no.info | 366 | 0.97 vs. **1.91** | 42.65 | **<0.001** |
| Gymnosperms | all | 47 | **1.05** vs. 0.47 | 8.21 | **0.0416** |
|  | harden | 36 | 0.74 vs. **1.85** | 4.20 | **0.0405** |
|  | no harden | 4 | 0.82 vs. 0.89 | 1.75 | 0.1863 |
|  | no.info | 10 | 0.16 vs. 0.81 | 0.42 | **0.5182** |
| Ferns | all | 31 | 0.081 vs. **0.29** | 11.78 | **<0.001** |
|  | harden | 5 | 0.020 vs. 0.11 | 3.26 | 0.0710 |
|  | no harden | - | - | - | - |
|  | no.info | 26 | 0.084 vs. **0.29** | 9.61 | **0.0019** |

**Table S3** Comparison of estimated evolutionary rates (σ^2^) between physiological tolerance (T_min_ and T_max_) and the margin of corresponding climatic niche (lower MTCM and upper MTWM).

| Group | Type | N | Evolutionary rate (σ^2^) | LRT | p |
| --- | --- | --- | --- | --- | --- |
| T_min_ vs. local MTCM | all | 582 | **306.92** vs. 26.08 | 1170.18 | **<0.001** |
| Angiosperms | all | 415 | **18.42** vs. 5.92 | 131.84 | **<0.001** |
|  | harden | 222 | **10.75** vs. 3.57 | 96.10 | **<0.001** |
|  | no harden | 84 | 1.33 vs. **2.60** | 10.86 | **<0.001** |
|  | no.info | 186 | 2.05 vs. **3.15** | 9.33 | **0.0022** |
| Gymnosperms | all | 71 | **2399.94** vs. 171.48 | 98.72 | **<0.001** |
|  | harden | 57 | **2961.31** vs. 212.21 | 79.11 | **<0.001** |
|  | no harden | 5 | 0.0069 vs. **1.35** | 21.78 | **<0.001** |
|  | no.info | 14 | **7.19** vs. 2.40 | 10.39 | **0.0013** |
| Ferns | all | 96 | 6.04 vs. 5.63 | 0.21 | 0.6499 |
|  | harden | - | - | - | - |
|  | no harden | - | - | - | - |
|  | no.info | 92 | 6.32 vs. 5.75 | 0.38 | 0.5362 |
| T_max_ vs. local MTWM | all | 895 | 1.79 vs. **2.92** | 53.09 | **<0.001** |
| Angiosperms | all | 813 | 1.90 vs. **3.06** | 55.47 | **<0.001** |
|  | harden | 469 | 1.35 vs. 1.32 | 0.07 | 0.7877 |
|  | no harden | 87 | 0.68 vs. **2.18** | 28.30 | **<0.001** |
|  | no.info | 370 | 0.95 vs. **3.87** | 181.83 | **<0.001** |
| Gymnosperms | all | 49 | 1.10 vs. **2.18** | 5.78 | **0.0163** |
|  | harden | 38 | 0.74 vs. **1.85** | 7.62 | **0.0058** |
|  | no harden | 4 | 0.82 vs. 0.89 | 0.02 | 0.8908 |
|  | no.info | 10 | 0.16 vs. **0.81** | 6.14 | **0.0132** |
| Ferns | all | 33 | 0.081 vs. **0.57** | 27.63 | **<0.001** |
|  | harden | - | - | - | - |
|  | no harden | - | - | - | - |
|  | no.info | 27 | 0.085 vs. **0.52** | 19.60 | **<0.001** |

**Table S4** Comparison of estimated evolutionary rates (σ^2^) between physiological tolerance (T_min_ and T_max_) and related local climatic niche (local MTCM and local MTWM).

**Table S5** Comparison of estimated absolute evolutionary rates (σ^2^) under three models (BM, OU, and LA) between physiological tolerance (T_min_ and T_max_) and related climatic niche (MTCM and MTWM). NA, no data. P values were derived from paired t-test. Best evolutionary models were present for each trait in the table.

| Group | Type | Absolute evolutionary rate | P (Paired t-test) | Best model for trait 1 | Best model for trait 2 |
| --- | --- | --- | --- | --- | --- |
| T_min_ vs. MTCM | all | **16.92** vs. 4.39 | **<0.001** | LA | LA |
|  | harden | **32.59** vs. 8.94 | **<0.001** | LA | LA |
|  | no harden | 0.34 vs. 0.62 | 0.3269 | OU | OU |
| Angiosperms | all | **2.07** vs. 1.27 | **0.0003** | LA | LA |
|  | harden | **1.78** vs. 0.98 | **<0.001** | LA | LA |
|  | no harden | 0.39 vs. **0.73** | **0.0143** | OU | LA |
|  | no.info | 0.45 vs. 0.38 | 0.0764 | LA | OU |
| Gymnosperms | all | **123.77** vs. 33.54 | **<0.001** | LA | LA |
|  | harden | **154.43** vs. 45.12 | **<0.001** | LA | LA |
|  | no harden | 0.012 vs. **0.33** | **0.0027** | BM | OU |
|  | no.info | 0.66 vs. 0.46 | 0.1348 | LA | LA |
| Ferns | all | **0.81** vs. 0.31 | **0.048** | LA | OU |
|  | harden | 0.024 vs. 0.045 | 0.3204 | BM | LA |
|  | no harden | 0.013 vs. 0.026 | 0.1304 | LA | BM |
|  | no.info | 0.83 vs. 0.32 | 0.0562 | LA | LA |
| T_max_ vs. MTWM | all | **0.036** vs. 0.033 | **0.0107** | OU | BM |
|  | harden | **0.041** vs. 0.030 | **<0.001** | OU | OU |
|  | no harden | 0.035 vs. 0.050 | 0.0721 | BM | OU |
| Angiosperms | all | 0.026 vs. 0.025 | 0.1302 | OU | LA |
|  | harden | **0.051** vs. 0.036 | **<0.001** | OU | OU |
|  | no harden | 0.25 vs. 0.28 | 0.1554 | LA | OU |
|  | no.info | **0.68** vs. 0.58 | **0.0015** | LA | LA |
| Gymnosperms | all | 0.28 vs. 0.27 | 0.4250 | OU | LA |
|  | harden | 0.24 vs. 0.25 | 0.4579 | OU | LA |
|  | no harden | 0.13 vs. 0.044 | 0.2421 | LA | BM |
|  | no.info | 0.10 vs. 0.088 | 0.9453 | BM | BM |
| Ferns | all | 0.035 vs. **0.071** | **0.0007** | LA | OU |
|  | harden | 0.017 vs. 0.035 | 0.4221 | BM | LA |
|  | no harden | - | - | - | - |
|  | no.info | 0.035 vs. **0.073** | **0.0001** | OU | OU |

Table S6. Comparison of estimated absolute evolutionary rates under three models (BM, OU, and LA) between cold tolerance and heat tolerance in both physiological tolerance (Tmin vs. Tmax) and climatic niche (MTCM vs. MTWM). NA, no data. P values were derived from paired t-test. Best evolutionary models were present for each trait in the table.

| Group | Absolute evolutionary rate | Paired t-test | Best model for trait 1 | Best model for trait 2 |
| --- | --- | --- | --- | --- |
| T_min_ vs. T_max_ | **0.64** vs. 0.25 | **<0.001** | OU | OU |
| Angiosperms | **0.47** vs. 0.28 | **0.0013** | OU | OU |
| Gymnosperms | **2.21** vs. 0.27 | **<0.001** | LA | OU |
| Ferns | **0.12** vs. 0.022 | **<0.001** | LA | LA |
| MTCM vs. MTWM | **2.32** vs. 1.15 | **<0.001** | LA | LA |
| Angiosperms | **1.16** vs. 0.87 | **<0.001** | LA | LA |
| Gymnosperms | **24.75** vs. 5.60 | **<0.001** | LA | LA |
| Ferns | **0.31** vs. 0.17 | **<0.001** | OU | LA |

Table S7. Comparison of estimated evolutionary rates (σ^2^) between cold tolerance and heat tolerance in both physiological tolerance (Tmin vs. Tmax) and climatic niche (MTCM vs. MTWM).

| Group | N | Evolutionary rate (σ^2^) | LRT | p |
| --- | --- | --- | --- | --- |
| T_min_ vs. T_max_ | 159 | **11.63** vs. 0.83 | 231.87 | **<0.001** |
| Angiosperms | 117 | **5.44** vs. 0.93 | 87.08 | **<0.001** |
| Gymnosperms | 23 | **51.97** vs. 0.98 | 64.04 | **<0.001** |
| Ferns | 19 | **0.84** vs. 0.05 | 30.52 | **<0.001** |
| MTCM vs. MTWM | 1245 | **9.39** vs. 2.01 | 664.80 | **<0.001** |
| Angiosperms | 1047 | **3.88** vs. 2.16 | 102.70 | **<0.001** |
| Gymnosperms | 95 | **78.25** vs. 2.10 | 237.83 | **<0.001** |
| Ferns | 103 | **1.89** vs. 0.44 | 62.18 | **<0.001** |

R codes for comparing overall evolutionary rates:

#Note: this script was developed by Adams (2013)

CompareRates.multTrait<-function(phy,x,TraitCov=T,ms.err=NULL,ms.cov=NULL){

library(MASS)

x<-as.matrix(x)

N<-nrow(x)

p<-ncol(x)

C<-vcv.phylo(phy)

C<-C[rownames(x),rownames(x)]

if (is.matrix(ms.err)){

ms.err<-as.matrix(ms.err[rownames(x),])}

if (is.matrix(ms.cov)){

ms.cov<-as.matrix(ms.cov[rownames(x),])}

#Cholesky decomposition function for diagonal-constrained VCV

build.chol<-function(b){

c.mat<-matrix(0,nrow=p,ncol=p)

c.mat[lower.tri(c.mat)] <- b[-1]

c.mat[p,p]<-exp(b[1])

c.mat[1,1]<-sqrt(sum((c.mat[p,])^2))

if(p>2){

for (i in 2:(p-1)){

c.mat[i,i]<-ifelse( (c.mat[1,1]^2-sum((c.mat[i,])^2) )>0,

sqrt(c.mat[1,1]^2-sum((c.mat[i,])^2)), 0)

}}

return(c.mat)

}

#Fit Rate matrix for all traits: follows code of L. Revell (evol.vcv)

a.obs<-colSums(solve(C))%*%x/sum(solve(C))

D<-matrix(0,N*p,p)

for(i in 1:(N*p)) for(j in 1:p) if((j-1)*N<i&&i<=j*N) D[i,j]=1.0

y<-as.matrix(as.vector(x))

one<-matrix(1,N,1)

R.obs<-t(x-one%*%a.obs)%*%solve(C)%*%(x-one%*%a.obs)/N

if (TraitCov==F) #for TraitCov = F

{ R.obs<-diag(diag(R.obs),p) }

#Calculate observed likelihood with or without measurement error

LLik.obs<-ifelse(is.matrix(ms.err)==TRUE,

-t(y-D%*%t(a.obs))%*%ginv((kronecker(R.obs,C)+ diag(as.vector(ms.err))))%*%(y-D%*%t(a.obs))/2-N*p*log(2*pi)/2-

determinant((kronecker(R.obs,C)+ diag(as.vector(ms.err))))$modulus[1]/2 ,

-t(y-D%*%t(a.obs))%*%ginv(kronecker(R.obs,C))%*%(y-D%*%t(a.obs))/2-N*p*log(2*pi)/2-

determinant(kronecker(R.obs,C))$modulus[1]/2

)

#Fit common rate for all traits; search over parameter space

sigma.mn<-mean(diag(R.obs)) #reasonable start value for diagonal

#Within-species measurement error matrix

if(is.matrix(ms.err)){m.e<-diag(as.vector(ms.err))}

#Within-species measurement error and trait covariation matrix

if (is.matrix(ms.err) && is.matrix(ms.cov)){

within.spp<-cbind(ms.err,ms.cov)

rc.label<-NULL

for (i in 1:p){ rc.label<-rbind(rc.label,c(i,i)) }

for (i in 1:p){

for (j in 2:p){ if (i!=j && i<j){rc.label<-rbind(rc.label,c(i,j))} }}

m.e<-NULL

for (i in 1:p){

tmp<-NULL

for (j in 1:p){

for (k in 1:nrow(rc.label)){

if(setequal(c(i,j),rc.label[k,])==T) {tmp<-cbind(tmp,diag(within.spp[,k]))}

}

}

m.e<-rbind(m.e,tmp)

}

}

#likelihood optimizer for no trait covariation

lik.covF<-function(sigma){

R<-matrix(0,nrow=p,ncol=p)

diag(R)<-sigma

LLik<-ifelse(is.matrix(ms.err)==TRUE,

-t(y-D%*%t(a.obs))%*%ginv((kronecker(R,C)+ m.e))%*%(y-D%*%t(a.obs))/2-N*p*log(2*pi)/2-

determinant((kronecker(R,C)+ m.e))$modulus[1]/2 ,

-t(y-D%*%t(a.obs))%*%ginv(kronecker(R,C))%*%(y-D%*%t(a.obs))/2-N*p*log(2*pi)/2-

determinant(kronecker(R,C))$modulus[1]/2

)

if (LLik == -Inf) { LLikk <- -1e+10 }

return(-LLik)

}

#likelihood optimizer with trait covariation

lik.covT<-function(sigma){

low.chol<-build.chol(sigma)

R<-low.chol%*%t(low.chol)

LLik<-ifelse(is.matrix(ms.err)==TRUE,

-t(y-D%*%t(a.obs))%*%ginv((kronecker(R,C)+ m.e))%*%(y-D%*%t(a.obs))/2-N*p*log(2*pi)/2-

determinant((kronecker(R,C)+ m.e))$modulus[1]/2 ,

-t(y-D%*%t(a.obs))%*%ginv(kronecker(R,C))%*%(y-D%*%t(a.obs))/2-N*p*log(2*pi)/2-

determinant(kronecker(R,C))$modulus[1]/2

)

if (LLik == -Inf) {LLikk <- -1e+10 }

return(-LLik)

}

##Optimize for no trait covariation

if (TraitCov==F)

{ model1<-optim(sigma.mn,fn=lik.covF,method="L-BFGS-B",lower=c(0.0))}

##Optimize with trait covariation

R.offd<-rep(0,(p*(p-1)/2))

if (TraitCov==T)

{model1<-optim(par=c(sigma.mn,R.offd),fn=lik.covT,method="Nelder-Mead")}

#### Assemble R.constrained

if (TraitCov==F){R.constr<-diag(model1$par,p)}

if (TraitCov==T){

chol.mat<-build.chol(model1$par)

R.constr<-chol.mat%*%t(chol.mat)}

if(model1$convergence==0)

message<-"Optimization has converged."

else

message<-"Optim may not have converged. Consider changing start value or lower/upper limits."

LRT<- (-2*((-model1$value-LLik.obs)))

LRT.prob<-pchisq(LRT, (p-1),lower.tail=FALSE) #df = Nvar-1

AIC.obs<- -2*LLik.obs+2*p+2*p #(2p twice: 1x for rates, 1x for anc. states)

AIC.common<- -2*(-model1$value)+2+2*p #(2*1: for 1 rate 2p for anc. states)

return(list(Robs=R.obs, Rconstrained=R.constr,Lobs=LLik.obs,Lconstrained=(-model1$value),LRTest=LRT,Prob=LRT.prob,

AICc.obs=AIC.obs,AICc.constrained=AIC.common,optimmessage=message))

}

R codes for calculating absolute evolutionary rates

rates<-function(tree,data,name){

names(data)<-name

WNfit <-fitContinuous (tree, data, model="white")

BMfit <-fitContinuous (tree, data, model="BM")

OUfit <-fitContinuous (tree, data, model="OU")

LAfit <-fitContinuous (tree, data, model="lambda")

AIC1 <-cbind.data.frame(WN=WNfit$opt$aic, BM=BMfit$opt$aic, OU=OUfit$opt$aic,LA=LAfit$opt$aic)

OU1tree<-rescale(tree,model='OU',alpha=OUfit$opt$alpha)

LA1tree<-rescale(tree,model='lambda',lambda=LAfit$opt$lambda)

BM1rec<-ace(data,tree,type="continuous",method="GLS",corStruct=corBrownian(1,tree))

OU1rec<-ace(data,OU1tree,type="continuous",method="GLS",corStruct=corBrownian(1,OU1tree))

LA1rec<-ace(data,LA1tree,type="continuous",method="GLS",corStruct=corBrownian(1,LA1tree))

BMace1 <- BM1rec$ace

OUace1 <- OU1rec$ace

LAace1 <- LA1rec$ace

Brtime <- branching.times(tree)

ACE <- cbind(Brtime,BMace1,OUace1,LAace1)

edge0 <- data.frame(tree$edge)

edge1 <- edge0[order(edge0$X2),]#order by latest node/tips

mrca<-edge1[edge1$X2<=length(tree$tip.label),]#only get the edges connect with tips

rownames(ACE)<-c((length(tree$tip.label)+1):(length(tree$tip.label)*2-1))#set prior nodes (from tips n+1 to 2n-1)

ACE2 <- data.frame(as.numeric(rownames(ACE)),ACE)

names(ACE2)[names(ACE2)=="as.numeric.rownames.ACE.."] <- "X1"

MRCA_ACE <- merge (ACE2, mrca, by ="X1") #merge by nodes

MRCA_ACE2<-MRCA_ACE[order(MRCA_ACE$X2),] # order tip from 1

RATE <-cbind(MRCA_ACE2,data)

RATE$Rate_BM <- with(RATE,abs(data-BMace1)/Brtime)

RATE$Rate_OU <- with(RATE,abs(data-OUace1)/Brtime)

RATE$Rate_LA <- with(RATE,abs(data-LAace1)/Brtime)

RATE2<-RATE[order(RATE$X2),]

rownames(RATE2) <- tree$tip.label

return(list(RATE2,AIC1))

}
